# Association of apolipoprotein E polymorphisms and dietary factors in colorectal cancer

**DOI:** 10.1038/sj.bjc.6605097

**Published:** 2009-05-19

**Authors:** M Mrkonjic, E Chappell, V V Pethe, M Manno, D Daftary, C M Greenwood, S Gallinger, B W Zanke, J A Knight, B Bapat

**Affiliations:** 1 Department of Laboratory Medicine and Pathobiology, University of Toronto, Toronto, ON, Canada M5G 1L5; 2 Samuel Lunenfeld Research Institute, Mount Sinai Hospital, Toronto, ON, Canada M5T 3L9; 3 Department of Pathology and Laboratory Medicine, Mount Sinai Hospital, Toronto, ON, Canada M5G 1X5; 4 Prosserman Centre for Health Research, Mount Sinai Hospital, Toronto, ON, Canada M5T 3L9; 5 Ontario Familial Colorectal Cancer Registry, Cancer Care Ontario, Toronto, ON, Canada M5G 3L7; 6 Dalla Lana School of Public Health, University of Toronto, Toronto, ON, Canada M5T 3M7; 7 Department of Surgery, Mount Sinai Hospital, Toronto, ON, Canada M5G 1X5; 8 Department of Surgery, University of Toronto, Toronto, ON, Canada M5G 1L5; 9 Ottawa Health Research Institute, Ottawa, ON, Canada K1Y 4E9

**Keywords:** CRC: colorectal cancer, ApoE: apolipoprotein E, MMR: mismatch repair, MSI-H: high-frequency microsatellite instability, MSS/L: microsatellite stable/low-frequency microsatellite instability, SNP: single nucleotide polymorphism

## Abstract

ApoE single nucleotide polymorphisms (SNPs) Cys112Arg (Epsilon-4), and Arg158Cys (Epsilon-2) have been implicated in cardiovascular and Alzheimer's disease, but their role in colorectal cancer (CRC) has not been extensively studied. We investigated whether ApoE polymorphisms alone or in combination with dietary factors selectively contribute to mismatch-repair (MMR) proficient (microsatellite stable/low or MSS/L) *vs* deficient (microsatellite unstable or MSI-H) CRCs. We carried out a case–control study with 906 CRC cases and 911 unaffected controls to examine the associations between ApoE polymorphisms and dietary factors and assessed their contribution to MSS/L and MSI-H CRCs. We used unconditional logistic regression to evaluate the associations between ApoE SNPs, tumour MSI status, and dietary factors after adjusting for age and sex. All statistical tests were two-sided. No significant differences in ApoE genotype frequencies were observed between CRC cases and unaffected controls. We observed that increased dietary intake of total fat, saturated fat, cholesterol, and red meat was significantly associated with CRC. Among non-ApoE4 carriers, 2–4 and >4 red meat servings/week were associated with developing MSS/L CRC (OR=1.51, 95% CI 1.10–2.07 and OR=1.80, 95% CI 1.30–2.48, respectively), whereas among ApoE4 allele carriers, four or more red meat servings/week were associated with MSI-H CRC (OR=4.62, 95% CI 1.20–17.77) when compared with the controls. ApoE isoforms modulate the risk of MSI-H and MSS/L CRCs among high red meat consumers.

Colorectal cancer (CRC) is a common malignancy and the second leading cause of cancer-related deaths in North America (Jemal *et al*, 2007). On the basis of mismatch-repair (MMR) functional status, CRCs can be subdivided into MMR-deficient, microsatellite unstable (MSI-H) and MMR-proficient, microsatellite stable (MSS) tumours. In addition to genetic alterations in tumour suppressor genes and oncogenes that are responsible for MSS tumours or defects in the DNA mismatch repair (Lynch and de la Chapelle, 2003), mutations and single nucleotide polymorphisms in genes involved in the metabolic pathways have also been implicated in CRC but to a lesser extent. Diets high in red and processed meat have been shown to increase risk of CRC, whereas diets rich in fruits, vegetables, and fibre lower CRC risk (Emmons *et al*, 2005; Ferrari *et al*, 2007). As dietary animal fat is a risk factor for CRC (Bautista *et al*, 1997; Slattery *et al*, 2000; Diergaarde *et al*, 2003; Wark *et al*, 2006), genetic alterations involved in the regulation of lipid transport and metabolism are potential susceptibility factors for colon cancer.

ApoE plays a multi-functional role in lipoprotein metabolism by acting as a high-affinity ligand for receptors of the LDL receptor family and serving as a cofactor in VLDL synthesis, as well as the hydrolysis of VLDL remnants in LDL production (Minihane *et al*, 2007). However, recent studies have identified ApoE functions other than lipid metabolism such as DNA synthesis, *β*-catenin localisation, cell proliferation, antioxidant abilities, angiogenesis, and metastasis that may play a role in CRC (Vogel *et al*, 1994; Grocott *et al*, 2001; Niemi *et al*, 2002; Cedazo-Minguez *et al*, 2003). The most widely studied polymorphisms in ApoE gene are Cys112Arg (rs429358) and Arg158Cys (rs7412), which create three distinct protein isoforms: ApoE2-epsilon2 (112Cys/158Cys), wild-type ApoE3-epsilon3 (112Cys/158Arg), and ApoE4-epsilon4 (112Arg/158Arg) (Weisgraber *et al*, 1981; Rall *et al*, 1982). ApoE4 is considered the high-risk isoform for chronic heart disease, Alzheimer's disease, and age-related cognitive decline (Corder *et al*, 1993; Wilson *et al*, 1996; Bretsky *et al*, 2003). However, the role of ApoE isoforms in CRC is not well established. All ApoE allelic variants are associated with distinct patterns of lipid transport and metabolism, and are shown to modulate enterohepatic metabolism of cholesterol and bile acids, which are implicated in promoting colorectal tumorigenesis (Debruyne *et al*, 2002; Minihane *et al*, 2007). Consequently, ApoE alleles are candidate risk factors for CRC. In a small study, ApoE4 was found to be protective from developing adenomas and carcinomas of the proximal colon (Kervinen *et al*, 1996), where the MSI-H subset of CRCs usually develop. A similar, but weak, inverse correlation of ApoE4 with proximal colonic location was reported among Australian CRC patients (Butler *et al*, 2001). MSI-H CRCs have distinct clinical and pathological manifestations from MSS CRCs such as: proximal colon location, mucinous histology, poor differentiation, Crohn-like reaction, tumour infiltrating lymphocytes (Alexander *et al*, 2001), and drug response (Elsaleh *et al*, 2001), and it is likely that distinct genetic and/or dietary risk factors contribute to these distinct CRC features. However, no study has evaluated the role of ApoE isoforms in CRC subtypes recognised by mismatch-repair deficient (MSI-H) and proficient (none or low microsatellite instability, MSS/L) profiles.

In this study, we investigated whether ApoE gene polymorphisms are associated with MSI-H and MSS/L CRC and whether they interact with dietary factors.

## Materials and methods

### Study subjects

The accrual of CRC patients and unaffected controls, the response rates, population characteristics, and ethnic backgrounds were described earlier (Mrkonjic *et al*, 2007; Raptis *et al*, 2007). Briefly, CRC cases and unaffected controls from the province of Ontario were obtained from the Ontario Familial Colorectal Cancer Registry (OFCCR). A total of 906 cases, aged 20–74 years, and diagnosed during the years 1997–2000, were identified for this study along with 911 unaffected controls. Ninety-five percent of participants in the OFCCR are of Caucasian ethnicity. Family history was collected by mailed questionnaires and was then used to construct pedigrees. Patients were classified by family risk. Further risk factor information was collected from two other mailed questionnaires and blood and tissue specimens were obtained. CRC patients were also stratified by family risk as described earlier by Cotterchio *et al* (2000). No apparent cases with familial adenomatous polyposis coli were included in the case series.

In addition to personal and family risk questionnaires, participants were also asked to complete a Food Frequency Questionnaire (FFQ). The FFQ was developed by the Epidemiology Program, Cancer Research Center of Hawaii and has been described earlier and validated (Cotterchio *et al*, 2008). Food frequency questionnaires were analysed using food composition databases that include values for macro- and micronutrients (Cotterchio *et al*, 2008). Data on saturated fat (grams), total fat (grams), and total cholesterol (milligrams), folacin (milligrams), and alcoholic beverages (per week) was obtained from the FFQ and was placed into quartiles. The frequency of red meat intake (servings per week) was obtained from a separate risk factor questionnaire and the values were also placed into quartiles. The research ethics boards of Mount Sinai Hospital and the University of Toronto approved all protocols.

### Molecular genetic analysis

#### Single nucleotide polymorphism genotyping

Genomic DNA was extracted from peripheral blood lymphocytes using phenol–chloroform or the Qiagen DNA extraction kit (Qiagen Inc., Montgomery County, MD, USA) as reported earlier (Mrkonjic *et al*, 2007; Raptis *et al*, 2007). Restriction Fragment Length Polymorphism (RFLP) was used to initially genotype the ApoE polymorphisms in CRC cases as described earlier (Gioia *et al*, 1998). Briefly, the initial PCR yields an amplicon of 485 bp of ApoE exon 4 containing both polymorphisms. The secondary, nested PCR yields a 300 bp product. The conditions of both PCRs, primer sequences, and restriction digestion analysis have been described earlier (Gioia *et al*, 1998). *Hha*I digestion of the nested amplicon generated unique patterns of restriction fragments depending on the original genotype of the individual. ApoE2 and ApoE3 lack *Hha*I restriction site at codon 112 and ApoE2 lacks the *Hha*I restriction site at codon 158. The restriction fragments were visualised through electrophoresis on a 4% agarose gel (Invitrogen, Burlington, Ontario, Canada).

With the availability of SNP genotyping arrays, we have genotyped CRC cases and unaffected controls for the ApoE4 SNP (rs429358) using the Illumina Golden Gate Assay (Illumina Inc., San Diego, CA, USA) as described earlier (Zanke *et al*, 2007). We used our previous RFLP genotyping of CRC cases for the rs429358 SNP to validate the genotyping calls. Because the ApoE2 SNP (rs7412) was not successfully genotyped using the Illumina Golden Gate Assay, we used TaqMan allelic discrimination assay to genotype the unaffected controls. The sequences of primers and probes are: forward primer CGCGATGCCGATGACCT, reverse primer GGCCCCGGCCTGGTAC, wild-type probe 6FAM-ACTGCCAGGCGCTTC-MGBNFQ, and variant probe VIC-ACTGCCAGGCACTTC-MGBNFQ. The conditions for the allelic discrimination reaction have been described earlier (Mrkonjic *et al*, 2007).

Additional independent validation for ApoE3/E4 was done on 55% of samples by TaqMan allelic discrimination assay as described earlier (MacLeod *et al*, 2001). The PCR conditions and primer/probe sequences have also been described earlier (MacLeod *et al*, 2001). All assays were run in 96-well polypropylene plates (Axygen Scientific, Union City, CA, USA) and the results were analysed using the Applied Biosystems 7900HT Sequence Detection system and accompanying software, SDS version 2.0 and/or 2.1 (Applied Biosystems, Foster City, CA, USA).

#### Tumour microsatellite instability (MSI) analysis

MSI analysis was performed as described earlier (Raptis *et al*, 2007). Briefly, paraffin-embedded colorectal tumour tissue of incident cases, and normal tissue from the same patient, were microdissected in areas with >70% cellularity in tumour and normal cell populations, respectively. MSI analysis was carried out using five or more microsatellite markers from the NCI recommended panel of 10 microsatellite markers; these markers consist of mononucleotides BAT-25, BAT-26, BAT-40, and BAT-34C4, dinucleotides D2S123, D5S346, ACTC, D18S55, and D10S197, and one penta-mono-tetra compound marker, MYC-L (Boland *et al*, 1998). The presence of altered/additional bands that resulted from the tumour PCR-amplified product, when compared with the matched normal colon PCR product, indicated MSI. Tumours were classified as MSI high (MSI-H, ⩾30% unstable markers among all markers tested), MSI low (MSI-L, <30% markers unstable) or microsatellite stable (MSS, no unstable markers) as per NCI recommended guidelines for MSI testing (Boland *et al*, 1998). MSI-L and MSS groups were combined into one group, hereafter referred to as simply ‘MSS/L’, for analysis purposes. Primer sequences and PCR amplification conditions for MSI testing have been described earlier (Raptis *et al*, 2007).

### Statistical analysis

The associations of ApoE2 and ApoE4 with colorectal cancer incidence, and with colorectal cancer subsets (MSI-H and MSS/L) were evaluated with a two-sided Pearson's *χ*^2^ test, in which a *P*-value of less than 0.05 was considered statistically significant. Unconditional logistic regression was also used to evaluate the association between ApoE2 and ApoE4 and CRC and its subsets, after adjusting for age and sex. The dietary differences between all CRC cases, or subsets stratified by tumour MSI status, and unaffected controls were evaluated using Pearson's *χ*^2^ or Fisher's exact test and with unconditional logistic regression, after adjusting for age and sex. Dietary differences between CRC subsets (MSI-H, MSS/L) and unaffected controls were also examined by ApoE4 and ApoE2 carrier status using unconditional logistic regression. In addition, we also examined differences in tumour location, stage and grade between MSI-H and MSS/L CRCs using Pearson's *χ*^2^ test and unconditional logistic regression. Chi-square and Fisher's exact tests, and logistic regression were performed with SAS version 9.0 (SAS Institute, Cary, NC, USA). All statistical tests were two-sided with a *P*-value <0.05 considered significant.

## Results

### Distribution of genotypes and alleles

We genotyped a total of 757 CRC cases for Cys112Arg (rs429358) and 779 CRC cases for Arg158Cys (rs7412) that give rise to ApoE4 and ApoE2 protein isoforms, respectively. We also genotyped 911 controls for ApoE2 and 864 controls for ApoE4 polymorphisms. Both polymorphisms are in Hardy–Weinberg equilibrium among the control population. A typical RFLP *Hha*I digest is shown in Supplementary Figure 1. The distribution of ApoE2 and ApoE4 genotypes between all cases and controls as well as cases stratified by tumour MSI status is shown in Table 1. The frequency of the ApoE4 allele among the CRC cases and unaffected controls was 13.6 and 14.8%, respectively. The frequency of the ApoE2 allele among the CRC cases and unaffected controls was 7.0 and 7.6%, respectively. We did not observe any statistically significant differences in genotype frequencies between all CRC cases or CRC subsets and unaffected controls (Table 1).

### Analysis of ApoE isoforms and dietary factors in CRC

We observed statistically significant associations of red meat consumption (*P*<0.001), total fat intake (*P*<0.001), saturated fat intake (*P*=0.002), and dietary cholesterol (*P*=0.003) with CRC; however, dietary folacin (*P*=0.14) or alcohol intake (*P*=0.38) did not show such association (Table 2). We did not observe any differences in dietary associations between CRC subsets stratified by tumour MSI status, except for the inverse association between drinking more than two alcoholic beverages per week and MSI-H CRCs (Table 3).

Among ApoE4 variant allele carriers we observed a statistically significant association between high red meat consumption (more than four servings per week) and the risk of MSI-H CRCs when compared with the controls (OR=4.62, 95% CI 1.20–17.77) (Table 4A). This association was not observed among non-ApoE4 carriers (Table 4B). However, among non-ApoE4 carriers a statistically significant association between red meat consumption (two or more servings of red meat per week) and the risk of MSS/L CRCs was observed when compared with the controls (for 2–4 red meat servings per week OR=1.51, 95% CI 1.10–2.07, and for more than four red meat servings per week OR=1.80, 95% CI 1.30–2.48) (Table 4B).

In contrast to ApoE4, we did not observe any statistically significant associations in ApoE2 carriers between dietary factors and CRC subsets (Table 5A). Similar to non-ApoE4 carriers, we observed statistically significant associations among non-ApoE2 carriers of red meat intake, total fat, saturated fat, and cholesterol intake with the risk of MSS/L and MSI-H CRCs when compared with the controls (Table 5B).

Among the CRC cases, we observed that MSI-H tumours occurred more proximally (*P*<0.001), and were statistically significantly associated with a higher tumour grade (*P*<0.001) when compared with the MSS/L tumours (Supplementary Table). We did not observe any differences in tumour stage between MSI-H and MSS/L CRCs.

## Discussion

This is the first study, to our knowledge, to examine the relationship between ApoE isoforms and MSI status in CRC. We observed that diets rich in fat or red meat increase the risk of CRC irrespective of tumour MSI status. We did not detect any differences in dietary folacin or alcohol intake between CRC cases and controls (Table 2), although it would appear that more than two alcoholic drinks per week might have a protective effect against MSI-H CRCs (Table 3). There were no associations between ApoE genotypes and CRC. When stratified by ApoE4 carrier status, red meat diet appears to increase the risk of MSI-H CRCs among ApoE4 carriers and MSS/MSI-L CRCs among non-ApoE4 carriers (Table 4). There is a borderline significant interaction between the ApoE4 allele, and red meat intake with respect to MSI-H CRCs (*P*=0.06), whereas no significant interaction is detected between ApoE4 and red meat with respect to MSS CRCs (*P*=0.13). Previous studies on red meat consumption and microsatellite instability have shown inconsistent results (Palli *et al*, 2001; Wu *et al*, 2001; Diergaarde *et al*, 2003). Although ApoE4 isoform and fat-rich diets are a high-risk combination for chronic heart disease and Alzheimer's disease, they seem to play a moderate role in the development of either CRC subtype (Table 4B). Associations with dietary factors in non-ApoE2 carriers are consistent with our overall findings (Table 5B). There were no significant associations among ApoE2 carriers, but the power was low (Table 5A).

The link between red meat intake and CRC has been known for many years (McKeown-Eyssen and Bright-See, 1984; Norat *et al*, 2005; Larsson and Wolk, 2006). Red meat contains high levels of haemoglobin, myoglobin, and cytochromes which are converted into denatured protein-hemes, hemichromes, and hemochromes, on cooking (Tappel, 2007). Free and coordinated hemes preferentially catalyse oxidative reactions that can damage DNA, proteins, lipids, and other nucleic acids (Tappel, 2007). In addition, heme also damages colonic mucosa and stimulates epithelial proliferation in animal models (Sesink *et al*, 1999). Red meat diet also leads to the formation of hetrocyclic amines and the endogenous formation of N-nitroso compounds (NOCs) (Cross and Sinha, 2004), which are known potent and organ-specific carcinogens (Magee and Barnes, 1956; Preussmann, 1984). Rats that were fed red meat diets showed significantly greater amounts of DNA single-strand breaks, double-strand breaks, and colonic mucous layer thinning than rats that were fed white meat diet (Toden *et al*, 2007).

ApoE has been well established to mediate the cellular uptake of lipoproteins by binding to the low-density lipoprotein receptor and the low-density lipoprotein receptor-related protein (LRP) (Lund *et al*, 1989; Yamada *et al*, 1992). However, ApoE also performs other crucial functions, and aberration of these functions may lead to cancer (Vogel *et al*, 1994; Grocott *et al*, 2001; Niemi *et al*, 2002; Cedazo-Minguez *et al*, 2003). ApoE binds heparin and proteoglycans with very high affinity and inhibits proliferation of several cell types such as: endothelial cells, lymphocytes, smooth muscle cells, and several types of tumour cells (Cardin *et al*, 1988; Ji *et al*, 1993; Browning *et al*, 1994; Vogel *et al*, 1994; Ishigami *et al*, 1998). *In vitro* studies suggest that treatment of colon cancer cell line HT29 with ApoE enhanced cell polarity by translocating *β*-catenin from the cytoplasm to cell–cell adhesion sites (Niemi *et al*, 2002). ApoE is a potent inhibitor of cell proliferation and *de novo* DNA synthesis (Vogel *et al*, 1994). Functional studies on ApoE isoforms showed that ApoE4, but not wild-type ApoE, significantly inhibits GSK-3*β* and increases the amount of active PKB (which further inactivates GSK-3*β*), leading to enhanced *β*-catenin translocation into the nucleus (Cedazo-Minguez *et al*, 2003). Nuclear *β*-catenin promotes transcription of genes involved in cell survival and division (Behrens *et al*, 1996; Molenaar *et al*, 1996; Pap and Cooper, 1998). These results would indicate that ApoE4 has pro-proliferative and anti-apoptotic properties.

Taken together, we propose that the selective effects of red meat diet on MSI-H CRC in ApoE4 carriers may be due to the anti-apoptotic and pro-proliferative effects of ApoE4. Heme and NOCs from red meat diets cause oxidative damage and DNA adduct formation, but rather than initiating cell cycle arrest and DNA repair, ApoE4-expressing cells induce pro-survival and proliferative signals. This would place a substantial strain on DNA repair machinery until a certain threshold level is reached beyond which the DNA repair mechanism is ineffective and genomic stability is compromised. Such cells would not undergo apoptosis even when extensive DNA damage is present. In addition, ApoE4 has poor antioxidant properties, compared with ApoE2, and combined with its proinflammatory functions may further exacerbate the tumourigenic process (Miyata and Smith, 1996; Jofre-Monseny *et al*, 2007). Conversely, ApoE2 and wild-type ApoE carriers would be able to better modulate aberrant cellular proliferation, inhibit oxidating and proinflammatory agents, allowing DNA repair machinery to more effectively repair DNA damage. Studies including fruits, vegetables, and antioxidant nutrients and their interaction with ApoE isoforms will further clarify this possible mechanism.

The main strength of our study is a well-characterised population. The frequencies of ApoE genotypes in our population are very similar to those of reports published earlier (Watson *et al*, 2003; Slattery *et al*, 2005; Bennet *et al*, 2007). We observed a strong association of MSI-H CRCs with female patients (Table 3), proximal colon location and higher tumour grades (Supplementary Table) consistent with reports published earlier (Thibodeau *et al*, 1993; Gryfe *et al*, 2000; Samowitz *et al*, 2001; Ribic *et al*, 2003). Limitations of our study are the small sample size of MSI-H CRCs and the low frequency of the ApoE2 polymorphism, which reduces our power to observe significant interactions among ApoE genotypes and dietary variables. Although the findings presented in this study need to be independently validated in another population, further characterisation of ApoE polymorphisms and dietary or environmental factors, may provide new insights into the gene–diet and gene–environment interactions and their contribution to incidence and progression of colon cancer or its subtypes.

## Figures and Tables

**Table 1:** Distribution of ApoE genotypes by cases and controls

**Variable**	**All cases *N* (%)**		**Controls *N* (%)**	***P*-value**	**OR (95% CI) all cases *vs* controls**	
*ApoE4*						
TT	568 (75.0)		634 (73.4)	0.56	1.00 (Referent)	
CT	172 (22.7)		204 (23.6)		0.94 (0.75, 1.19)	
CC	17 (2.2)		26 (3.0)		0.73 (0.39, 1.36)	
				
*ApoE2*						
TT	673 (86.4)		779 (85.5)	0.69	1.00 (Referent)	
CT	103 (13.2)		126 (13.8)		0.95 (0.72, 1.25)	
CC	3 (0.4)		6 (0.7)		0.58 (0.14, 2.32)	
						
	**MSS/L N (%)**	**MSI-H N (%)**			**OR (95% CI) MSS/L *vs* controls**	**OR (95% CI) MSI-H *vs* controls**
*ApoE4*						
TT	391 (75.0)	63 (73.3)	634 (73.4)	0.85	1.00 (Referent)	1.00 (Referent)
CT	119 (22.8)	20 (23.3)	204 (23.6)		0.95 (0.73, 1.23)	0.99 (0.58, 1.67)
CC	11 (2.1)	3 (3.5)	26 (3.0)		0.69 (0.34, 1.40)	1.16 (0.34, 3.94)
						
*ApoE2*						
TT	541 (86.8)	94 (84.7)	779 (85.5)	0.70	1.00 (Referent)	1.00 (Referent)
CT/CC	82 (13.2)	17 (15.3)	132 (14.5)		0.89 (0.66, 1.20)	1.07 (0.62, 1.85)

ApoE2 represents ApoE protein with 112Cys/158Cys residues, ApoE4 represents ApoE protein with 112Arg/158Arg residues, MSI-H=high frequency microsatellite instability, MSS/L=microsatellite stable/low-frequency microsatellite instability, homozygous wild-type allele carriers were considered as the referent groups.

**Table 2:** Association of sex, age at diagnosis, red meat intake (servings per week), total fat intake (grams), saturated fat intake (grams), cholesterol intake (milligrams), dietary folacin (milligrams) and alcohol intake (drinks/week) between CRC cases and controls

**Variable**	**Case *N* (%)**	**Control *N* (%)**	**Total *N* (%)**	***P*-value**	**OR (95% CI)**
*Sex*					
Female	441 (48.7)	402 (46.2)	843 (47.5)	0.30	1.00
Male	465 (51.3)	468 (53.8)	933 (52.5)		0.91 (0.75, 1.09)
					
*Age at diagnosis*					
<50	113 (12.5)	77 (8.9)	190 (10.7)	0.01	1.00 (Referent)
50–59	257 (28.4)	299 (34.4)	556 (31.3)		0.59 (0.42, 0.82)
60–69	402 (44.4)	372 (42.8)	774 (43.6)		0.74 (0.53, 1.02)
70+	133 (14.7)	121 (13.9)	254 (14.3)		0.75 (0.51, 1.10)
					
*Red meat consumption/week*					
0.0–2.0	247 (27.9)	317 (36.8)	564 (32.3)	<0.001	1.00 (Referent)
2.1–3.0	186 (21.0)	159 (18.5)	345 (19.7)		1.50 (1.15, 1.97)
3.1–5.0	225 (25.4)	205 (23.8)	430 (24.6)		1.41 (1.09, 1.81)
>5.0	228 (25.7)	180 (20.9)	408 (23.4)		1.63 (1.26, 2.10)
					
*Total fat intake (grams)*					
0–50	175 (19.3)	222 (25.5)	397 (22.4)	<0.001	1.00 (Referent)
>50–70	262 (28.9)	244 (28.0)	506 (28.5)		1.36 (1.05, 1.77)
>70–90	197 (21.7)	205 (23.6)	402 (22.6)		1.22 (0.92, 1.61)
>90	272 (30.0)	199 (22.9)	471 (26.5)		1.73 (1.32, 2.27)
					
*Saturated fat intake (grams)*					
0–15	200 (22.1)	254 (29.2)	454 (25.6)	<0.01	1.00 (Referent)
>15–20	206 (22.7)	204 (23.4)	410 (23.1)		1.28 (0.98, 1.68)
>20–27	234 (25.8)	203 (23.3)	437 (24.6)		1.46 (1.12, 1.91)
>27	266 (29.4)	209 (24.0)	475 (26.7)		1.62 (1.25, 2.09)
					
*Dietary cholesterol (milligrams)*					
0–150	169 (18.7)	216 (24.8)	385 (21.7)	<0.01	1.00 (Referent)
>150–220	265 (29.2)	262 (30.1)	527 (29.7)		1.29 (0.99, 1.68)
>220–290	222 (24.5)	202 (23.2)	424 (23.9)		1.40 (1.06, 1.85)
>290	250 (27.6)	190 (21.8)	440 (24.8)		1.68 (1.28, 2.22)
					
*Dietary folacin (miligrams)*					
0–250	201 (22.2)	214 (24.6)	415 (23.4)	0.14	1.00 (Referent)
>250–370	264 (29.1)	225 (25.9)	489 (27.5)		1.25 (0.96, 1.62)
>370–520	232 (25.6)	203 (23.3)	435 (24.5)		1.22 (0.93, 1.59)
>520	209 (23.1)	228 (26.2)	437 (24.6)		0.98 (0.75, 1.28)
					
*Alcoholic drinks/week*					
None	224 (25.9)	194 (23.2)	418 (24.6)	0.40	1.00 (Referent)
0.1–2.0	196 (22.6)	208 (24.9)	404 (23.7)		0.82 (0.62, 1.07)
2.1–7.0	228 (26.3)	236 (28.2)	464 (27.3)		0.84 (0.64, 1.09)
>7.0	218 (25.2)	198 (23.7)	416 (24.4)		0.95 (0.73, 1.25)

CI=confidence interval; OR=odds ratios adjusted for age and sex. All samples with unavailable data have been omitted from the analyses. Female participants, participants with age <50 along with lowest dietary groups have been used as the referent goups.

**Table 3:** Association of sex, age at diagnosis, and dietary factors between CRC cases stratified by MSI tumour status and controls

**Variable**	**Controls *N* (%)**	**MSS/L *N* (%)**	**MSI-H *N* (%)**	**Total *N* (%)**	***P*-value**	**OR (95% CI) MSS/L *vs* controls**	**OR (95% CI) MSI-H *vs* controls**
*Sex*							
Female	402 (46.2)	302 (47.2)	71 (63.4)	775 (47.8)	<0.01	1.00 (Referent)	1.00 (Referent)
Male	468 (53.8)	338 (52.8)	41 (36.6)	847 (52.2)		0.96 (0.78, 1.18)	0.50 (0.33, 0.75)
							
*Age at diagnosis*							
<50	77 (8.9)	75 (11.7)	15 (13.4)	167 (10.3)	0.01	1.00 (Referent)	1.00 (Referent)
50–59	299 (34.4)	187 (29.2)	28 (25.0)	514 (31.7)		0.64 (0.44, 0.93)	0.48 (0.24, 0.94)
60–69	372 (42.8)	282 (44.1)	42 (37.5)	696 (42.9)		0.78 (0.55, 1.11)	0.58 (0.31, 1.10)
70+	121 (13.9)	96 (15.0)	27 (24.1)	244 (15.1)		0.81 (0.54, 1.23)	1.15 (0.57, 2.29)
							
*Red meat consumption/week*							
0.0–2.0	317 (36.8)	170 (27.2)	30 (27.3)	517 (32.4)	<0.01	1.00 (Referent)	1.00 (Referent)
2.1–3.0	159 (18.5)	134 (21.5)	25 (22.7)	318 (19.9)		1.57 (1.17, 2.11)	1.66 (0.95, 2.92)
3.1–5.0	205 (23.8)	162 (26.0)	29 (26.4)	396 (24.8)		1.47 (1.12, 1.95)	1.49 (0.87, 2.56)
>5.0	180 (20.9)	158 (25.3)	26 (23.6)	364 (22.8)		1.64 (1.23, 2.17)	1.53 (0.88, 2.66)
							
*Red meat consumption/week (tertiles)*							
0.0–2.0	317 (36.8)	170 (27.2)	30 (27.3)	517 (32.4)	<0.001	1.00 (Referent)	1.00 (Referent)
2.1–4.0	293 (34.0)	227 (36.4)	49 (44.5)	569 (35.7)		1.44 (1.12, 1.86)	1.77 (1.09, 2.86)
>4.0	251 (29.2)	227 (36.4)	31 (28.2)	509 (31.9)		1.69 (1.30, 2.18)	1.31 (0.77, 2.21)
							
*Total fat intake*							
0–50	222 (25.5)	122 (19.1)	20 (17.9)	364 (22.4)	0.01	1.00 (Referent)	1.00 (Referent)
>50–70	244 (28.0)	189 (29.5)	30 (26.8)	463 (28.5)		1.41 (1.05, 1.89)	1.36 (0.75, 2.47)
>70–90	205 (23.6)	140 (21.9)	27 (24.1)	372 (22.9)		1.24 (0.91, 1.69)	1.46 (0.80, 2.69)
>90	199 (22.9)	189 (29.5)	35 (31.3)	423 (26.1)		1.73 (1.28, 2.33)	1.95 (1.09, 3.49)
							
*Total fat intake (tertiles)*							
0–57	315 (36.2)	183 (28.6)	31 (27.7)	529 (32.6)	<0.01	1.00 (Referent)	1.00 (Referent)
>57–83	300 (34.5)	226 (35.3)	37 (35.3)	563 (34.7)		1.30 (1.01, 1.67)	1.25 (0.76, 2.07)
>83	255 (29.3)	231 (36.1)	44 (39.3)	530 (32.7)		1.56 (1.21, 2.01)	1.75 (1.08, 2.86)
							
*Saturated fat intake*							
0–15	254 (29.2)	140 (21.9)	22 (19.6)	416 (25.6)	<0.01	1.00 (Referent)	1.00 (Referent)
>15–20	204 (23.4)	149 (23.3)	21 (18.8)	374 (23.1)		1.33 (0.99, 1.78)	1.19 (0.64, 2.22)
>20–27	203 (23.3)	166 (25.9)	35 (31.3)	404 (24.9)		1.48 (1.11, 1.98)	1.99 (1.13, 3.50)
>27	209 (24.0)	185 (28.9)	34 (30.4)	428 (26.4)		1.61 (1.21, 2.14)	1.88 (1.07, 3.31)
							
*Saturated fat intake (tertiles)*							
0–18	384 (44.1)	223 (34.8)	36 (32.1)	643 (39.6)	<0.001	1.00 (Referent)	1.00 (Referent)
>18–25	230 (26.4)	197 (30.8)	29 (25.9)	456 (28.1)		1.47 (1.15, 1.90)	1.34 (0.80, 2.25)
>25	256 (29.4)	220 (34.4)	47 (42.0)	523 (32.2)		1.48 (1.16, 1.89)	1.96 (1.23, 3.11)
							
*Dietary cholesterol*							
0–150	216 (24.8)	114 (17.8)	24 (21.4)	354 (21.8)	0.02	1.00 (Referent)	1.00 (Referent)
>150–220	262 (30.1)	187 (29.2)	30 (26.8)	479 (29.5)		1.35 (1.01, 1.82)	1.03 (0.58, 1.82)
>220–290	202 (23.2)	168 (26.3)	25 (22.3)	395 (24.4)		1.58 (1.16, 2.14)	1.11 (0.62, 2.01)
>290	190 (21.8)	171 (26.7)	33 (29.5)	394 (24.3)		1.71 (1.25, 2.32)	1.56 (0.89, 2.74)
							
*Dietary cholesterol (tertiles)*							
0–180	334 (38.4)	190 (29.7)	42 (37.5)	566 (34.9)	<0.01	1.00 (Referent)	1.00 (Referent)
>180–260	267 (30.7)	219 (34.2)	29 (25.9)	515 (31.8)		1.44 (1.12, 1.86)	0.86 (0.52, 1.42)
>260	269 (30.9)	231 (36.1)	41 (36.6)	541 (33.4)		1.51 (1.18, 1.94)	1.21 (0.77, 1.92)
							
*Dietary folacin*							
0–250	214 (24.6)	137 (21.4)	24 (21.4)	375 (23.1)	0.34	1.00 (Referent)	1.00 (Referent)
>250–370	225 (25.9)	192 (30.0)	32 (28.6)	449 (27.7)		1.33 (1.00, 1.78)	1.27 (0.72, 2.22)
>370–520	203 (23.3)	165 (25.8)	27 (24.1)	395 (24.4)		1.27 (0.94, 1.71)	1.19 (0.66, 2.12)
>520	228 (26.2)	146 (22.8)	29 (25.9)	403 (24.8)		1.00 (0.74, 1.35)	1.13 (0.64, 2.01)
							
*Dietary folacin (tertiles)*							
0–290	299 (34.4)	202 (31.6)	38 (33.9)	539 (33.2)	0.37	1.00 (Referent)	1.00 (Referent)
>290–460	277 (31.8)	235 (36.7)	35 (31.3)	547 (33.7)		1.26 (0.98, 1.61)	0.99 (0.61, 1.62)
>460	294 (33.8)	203 (31.7)	39 (34.8)	536 (33.0)		1.02 (0.79, 1.32)	1.04 (0.65, 1.68)
							
*Alcoholic drinks/week*							
None	194 (23.2)	146 (23.9)	39 (36.1)	379 (24.4)	0.11	1.00 (Referent)	1.00 (Referent)
0.1–2.0	208 (24.9)	140 (22.9)	25 (23.1)	373 (24.0)		0.89 (0.66, 1.21)	0.60 (0.35, 1.02)
2.1–7.0	236 (28.2)	171 (28.0)	23 (21.3)	430 (27.7)		0.96 (0.72, 1.29)	0.48 (0.28, 0.84)
>7.0	198 (23.7)	154 (25.2)	21 (19.4)	373 (24.0)		1.03 (0.77, 1.40)	0.53 (0.30, 0.93)

**Table 4:** Association of dietary factors between CRC cases stratified by MSI tumour status and controls by ApoE4 allele carrier status

**Variable**	**Controls *N* (%)**	**MSS/L *N* (%)**	**MSI-H *N* (%)**	**OR (95% CI) MSS/L *vs* controls**	**OR (95% CI) MSI-H *vs* controls**
*(A) ApoE4 carriers*					
Red meat consumption/week					
0.0–2.0	81 (35.8)	42 (33.3)	3 (13.6)	1.00 (Referent)	1.00 (Referent)
2.1–4.0	78 (34.5)	45 (35.7)	9 (40.9)	1.13 (0.67, 1.91)	3.25 (0.84, 12.56)
>4.0	67 (29.7)	39 (31.0)	10 (45.5)	1.16 (0.67, 2.02)	4.62 (1.20, 17.77)
Dietary cholesterol (milligrams)					
0–180	89 (38.7)	42 (32.3)	9 (39.1)	1.00 (Referent)	1.00 (Referent)
>180–260	72 (31.3)	38 (29.2)	7 (30.4)	1.24 (0.71, 2.15)	1.11 (0.38, 3.18)
>260	69 (30.0)	50 (38.5)	7 (30.4)	1.87 (1.08, 3.26)	1.22 (0.41, 3.69)
Saturated fat intake (grams)					
0–18	104 (45.2)	52 (40.0)	8 (34.8)	1.00 (Referent)	1.00 (Referent)
>18–25	58 (25.2)	37 (28.5)	5 (21.7)	1.37 (0.80, 2.34)	1.22 (0.38, 3.96)
>25	68 (29.6)	41 (31.5)	10 (43.5)	1.36 (0.80, 2.32)	2.31 (0.83, 6.40)
Total fat intake (grams)					
0–57	82 (35.6)	39 (30.0)	5 (21.7)	1.00 (Referent)	1.00 (Referent)
>57–83	83 (36.1)	50 (38.5)	9 (39.1)	1.37 (0.81, 2.32)	2.01 (0.64, 6.36)
>83	65 (28.3)	41 (31.5)	9 (39.1)	1.58 (0.89, 2.81)	3.01 (0.91, 9.95)
					
*(B) Non-ApoE4 carriers*					
Red meat consumption/week					
0.0–2.0	234 (37.2)	101 (26.5)	21 (33.9)	1.00 (Referent)	1.00 (Referent)
2.1–4.0	215 (34.2)	140 (36.8)	25 (40.3)	1.51 (1.10, 2.07)	1.33 (0.72, 2.46)
>4.0	180 (28.6)	140 (36.8)	16 (25.8)	1.80 (1.30, 2.48)	1.03 (0.52, 2.05)
Dietary cholesterol (milligrams)					
0–180	242 (38.2)	109 (27.9)	25 (39.7)	1.00 (Referent)	1.00 (Referent)
>180–260	194 (30.6)	142 (36.3)	14 (22.2)	1.64 (1.20, 2.25)	0.81 (0.41, 1.62)
>260	198 (31.2)	140 (35.8)	24 (38.1)	1.58 (1.14, 2.18)	1.58 (0.85, 2.93)
Saturated fat intake (grams)					
0–18	277 (43.7)	128 (32.7)	20 (31.8)	1.00 (Referent)	1.00 (Referent)
>18–25	171 (27.0)	125 (32.0)	15 (23.8)	1.60 (1.17, 2.19)	1.38 (0.68, 2.80)
>25	186 (29.3)	138 (35.3)	28 (44.4)	1.61 (1.18, 2.20)	2.80 (1.50, 5.25)
Total fat intake (grams)					
0–57	230 (36.3)	108 (27.6)	19 (30.2)	1.00 (Referent)	1.00 (Referent)
>57–83	216 (34.1)	134 (34.3)	18 (28.6)	1.34 (0.98, 1.84)	1.14 (0.58, 2.26)
>83	188 (29.6)	149 (38.1)	26 (41.3)	1.71 (1.24, 2.37)	2.29 (1.20, 4.38)

CI=confidence interval; MSI-H=high frequency microsatellite instability; MSS/L=microsatellite stable/low-frequency microsatellite instability; OR=odds ratios adjusted for age and sex. All samples with unavailable data have been omitted from the analyses. Lowest dietary groups have been used as the referent groups.

**Table 5:** Association of dietary factors between CRC cases stratified by MSI tumour status and controls by ApoE2 allele carrier status

**Variable**	**Controls *N* (%)**	**MSS/L *N* (%)**	**MSI-H *N* (%)**	**OR (95% CI) MSS/L *vs* controls**	**OR (95% CI) MSI-H *vs* controls**
*(A) ApoE2 carriers*					
Red meat consumption/week					
0.0–2.0	40 (30.3)	17 (21.0)	4 (25.0)	1.00 (Referent)	1.00 (Referent)
2.1–4.0	47 (35.6)	30 (37.0)	8 (50.0)	1.40 (0.67, 2.93)	1.48 (0.40, 5.45)
>4.0	45 (34.1)	34 (42.0)	4 (25.0)	1.76 (0.85, 3.65)	0.87 (0.20, 3.80)
Dietary cholesterol (milligrams)					
0–180	40 (30.3)	25 (30.5)	8 (47.1)	1.00 (Referent)	1.00 (Referent)
>180–260	45 (34.1)	26 (31.7)	3 (17.6)	0.95 (0.47, 1.93)	0.39 (0.09, 1.59)
>260	47 (35.6)	31 (37.8)	6 (35.3)	1.14 (0.57, 2.27)	0.79 (0.24, 2.53)
Saturated fat intake (grams)					
0–18	50 (37.9)	26 (31.7)	7 (41.2)	1.00 (Referent)	1.00 (Referent)
>18–25	32 (24.2)	29 (35.4)	5 (29.4)	1.84 (0.90, 3.73)	1.38 (0.39, 4.89)
>25	50 (37.9)	27 (32.9)	5 (29.4)	1.12 (0.57, 2.21)	0.86 (0.25, 2.98)
Total fat intake (grams)					
0–57	40 (30.3)	26 (31.7)	8 (47.1)	1.00 (Referent)	1.00 (Referent)
>57–83	44 (33.3)	25 (30.5)	2 (11.7)	0.85 (0.42, 1.73)	0.24 (0.05, 1.20)
>83	48 (36.4)	31 (37.8)	7 (41.2)	1.08 (0.54, 2.13)	0.91 (0.29, 2.84)
					
*(B) Non-ApoE2 carriers*					
Red meat consumption/week					
0.0–2.0	288 (37.5)	149 (28.3)	26 (28.0)	1.00 (Referent)	1.00 (Referent)
2.1–4.0	263 (34.2)	192 (36.4)	40 (43.0)	1.41 (1.07, 1.85)	1.76 (1.04, 2.98)
>4.0	217 (28.3)	186 (35.3)	27 (29.0)	1.68 (1.27, 2.23)	1.51 (0.85, 2.69)
Dietary cholesterol (milligrams)					
0–180	309 (39.7)	162 (29.9)	33 (35.1)	1.00 (Referent)	1.00 (Referent)
>180–260	239 (30.7)	185 (34.2)	26 (27.7)	1.52 (1.16, 2.00)	1.19 (0.68, 2.06)
>260	231 (29.6)	194 (35.9)	35 (37.2)	1.67 (1.26, 2.21)	1.89 (1.11, 3.23)
Saturated fat intake (grams)					
0–18	357 (45.8)	189 (34.9)	28 (29.8)	1.00 (Referent)	1.00 (Referent)
>18–25	207 (26.6)	164 (30.3)	24 (25.5)	1.54 (1.17, 2.02)	1.67 (0.94, 2.98)
>25	215 (27.6)	188 (34.8)	42 (44.7)	1.72 (1.31, 2.26)	3.26 (1.92, 5.54)
Total fat intake (grams)					
0–18	289 (37.1)	151 (28.0)	23 (24.5)	1.00 (Referent)	1.00 (Referent)
>18–25	273 (35.0)	195 (36.0)	34 (36.2)	1.39 (1.06, 1.83)	1.76 (1.00, 3.09)
>25	217 (27.9)	195 (36.0)	37 (39.3)	1.83 (1.37, 2.43)	2.92 (1.65, 5.18)

CI=confidence interval; MSI-H=high frequency microsatellite instability; MSS/L=microsatellite stable/ low frequency microsatellite instability; OR=odds ratios adjusted for age and sex. All samples with unavailable data have been omitted from the analyses. Lowest dietary groups have been used as the referent groups.
