# Supplementary material for: Association of apolipoprotein E polymorphisms and dietary factors in colorectal cancer
Source: Br J Cancer. 2009 May 19;100(12):1966–74. doi: 10.1038/sj.bjc.6605097 (PMC2714237; doi:10.1038/sj.bjc.6605097)
Supplement: Supplementary Figure 1 [file 6605097x1.ppt]

## Slide 1
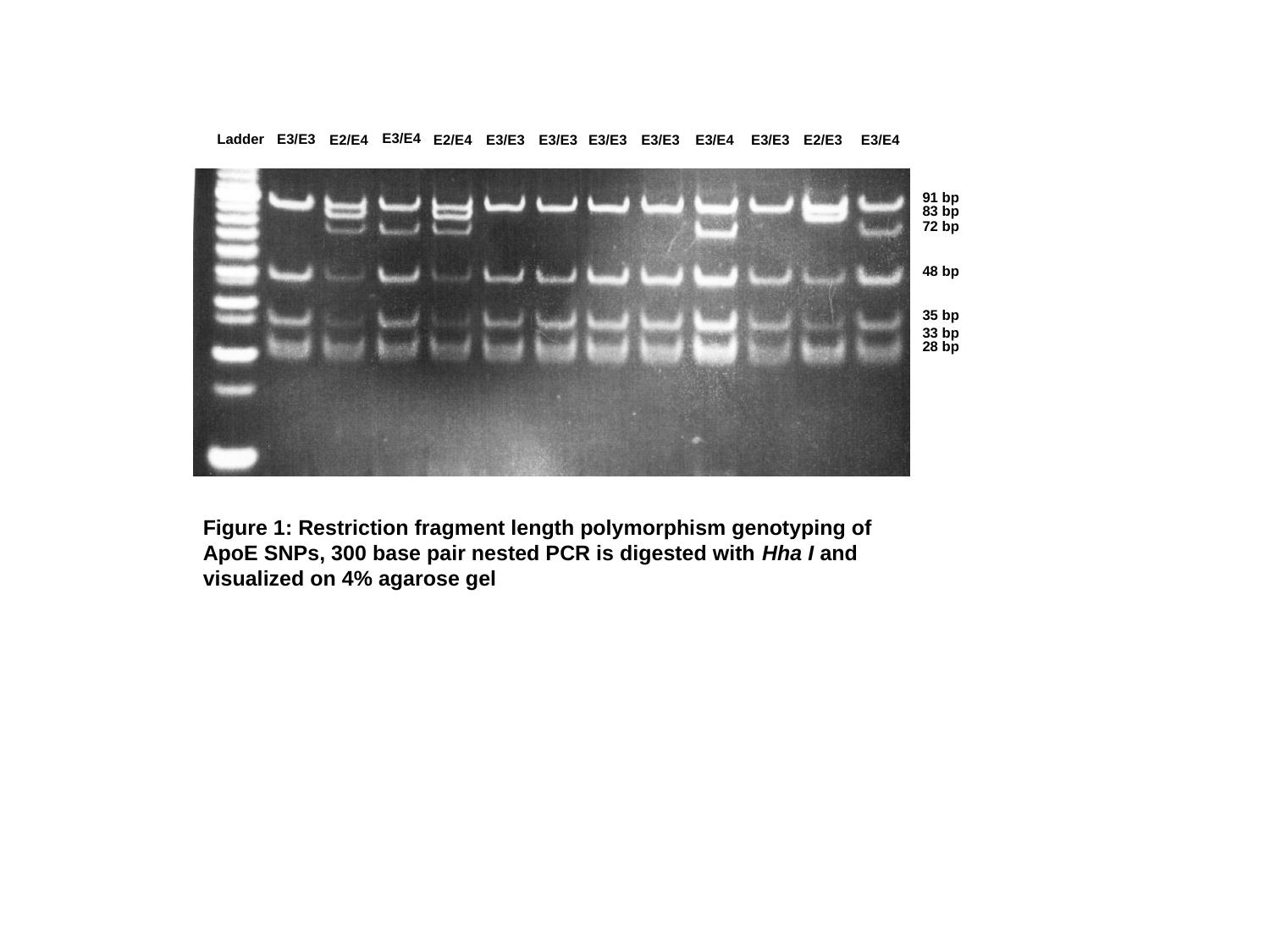

E3/E4
E3/E3
E2/E4
E3/E3
E3/E3
E3/E3
E3/E3
E3/E4
E3/E3
E2/E3
E3/E4
E2/E4
Ladder
91 bp
83 bp
72 bp
48 bp
35 bp
33 bp
28 bp
Figure 1: Restriction fragment length polymorphism genotyping of ApoE SNPs, 300 base pair nested PCR is digested with Hha I and visualized on 4% agarose gel
