# Supplementary material for: Association of apolipoprotein E polymorphisms and dietary factors in colorectal cancer
Source: Br J Cancer. 2009 May 19;100(12):1966–74. doi: 10.1038/sj.bjc.6605097 (PMC2714237; doi:10.1038/sj.bjc.6605097)
Supplement: Supplementary Table [file 6605097x2.doc]

**Supplemental Table:** Association of clinical and pathological tumour features as well as dietary factors by MSI tumour status

|  | **MSI Status** | |  |  |  |
| --- | --- | --- | --- | --- | --- |
| **Variable** | **MSI-H** | **MSS/L** | **Total** | **P-Value** | **OR (95% CI)** |
|  | **N (%)** | **N (%)** | **N (%)** |  |  |
| **Tumour Location** |  |  |  |  |  |
| Proximal | 86 (63.7) | 209 (26.9) | 295 (32.3) | <0.001 | 1.00 (Referent) |
| Distal | 20 (14.8) | 431 (55.4) | 451 (49.4) |  | 0.11 (0.07, 0.19) |
| Other | 29 (21.5) | 138 (17.7) | 167 (18.3) |  | 0.51 (0.32, 0.82) |
| **Tumour Grade** |  |  |  |  |  |
| 1 | 15 (12.8) | 67 (11.0) | 82 (11.3) | <0.001 | 1.00 (Referent) |
| 2 | 63 (53.8) | 434 (71.5) | 497 (68.6) |  | 0.65 (0.35, 1.20) |
| 3 | 30 (25.6) | 57 (9.4) | 87 (12.0) |  | 2.35 (1.15, 4.80) |
| Not determined | 9 (7.7) | 49 (8.1) | 58 (8.0) |  | 0.82 (0.33, 2.03) |
| **Tumour Stage** |  |  |  |  |  |
| 1 | 5 (4.3) | 40 (6.6) | 45 (6.2) | 0.19 | 1.00 (Referent) |
| 2 | 19 (16.2) | 114 (18.8) | 133 (18.4) |  | 1.33 (0.47, 3.81) |
| 3 | 76 (65.0) | 368 (60.6) | 444 (61.3) |  | 1.65 (0.63, 4.32) |
| 4 | 13 (11.1) | 41 (6.8) | 54 (7.5) |  | 2.54 (0.83, 7.77) |
| Not determined | 4 (3.4) | 44 (7.2) | 48 (6.6) |  | 0.73 (0.18, 2.90) |

MSI-H = high frequency microsatellite instability, MSS/L = microsatellite stable/ low frequency microsatellite instability, OR = odds ratios adjusted for age and sex, CI = confidence interval, P-values obtained from two-sided Pearson’s chi-square tests. All samples with unavailable data have been omitted from the analyses.
